# History of a Case in Which Excision of a Portion of the Lower Jaw Was Practised by M. Dupuytren, to Obtain the Re-Union of Fragments of This Bone, Resulting from a Fracture of It with Loss of Substance and Want of Consolidation, Consequent on a Wound by a Fire-Arm

**Published:** 1820-09

**Authors:** L. J. Sanson

**Affiliations:** Doctor in Surgery of the Faculty of Paris.


FOR THE LONDON MEDICAL AND PHYSICAL JOURNAL.

*History of a Case in which Excision of a Portion of the Lower Jaw was practised by* M. Dupuytren, *to obtain the Re-union of Fragments of this Bone, resulting from a Fracture of it with loss of Substance and want of Consolidation, consequent on a Wound by a Fire-arm.* Related by L. J. Sanson, Doctor in Surgery of the Faculty of Paris.

Mr. de R—, aide-de-camp to Lieutenant-general Count Woronzoff, received, in the affair at Brienne, in 1814, as he was advancing to protect the retreat of the Russians, whom the French had dislodged from the castle, a charge from a fire-arm, which brought him to the ground. He was immediately taken up, and carried to the surgeons of his regiment.

A ball had traversed the superior part of the neck and the inferior part of the face, in a direction upwards and from left to right. The wound made by the entry of the ball was immediately below the base and the angle of the jaw, before and close to the external carotid artery, and above the os hyoides. The wound made by the escape of the ball was on the opposite side, the right, higher, and in front of the insertion of the masseter muscle: it corresponded so exactly with the body of the jaw, that the ball, in passing out, must have separated the body and the condyloid apophysis, or rising branch, of the bone from each other. The common teguments, the muscles situate on the lateral parts of the neck, and the base of the tongue, were then traversed by the ball, and the lower jaw had suffered comminutive fracture. There were no signs of any lesion of the epiglottis or os hyoides.

The difficulty with which Mr. de R. expresses himself in French, prevented any thing being collected respecting the immediate consequences of the accident; beyond this,—that he in the first instance lost a large quantity of blood; that a bandage was applied; that for some time deglutition and respiration were effected with very great difficulty; that, after the separation of the sloughs, an abundant suppuration took place; and, lastly, that after six months' care, and the spontaneous issue or the extraction of a great number of splinters, the wounds were cicatrized, without the fracture of the jaw having become consolidated.

Judging from the number and the volume of the splinters which the patient has preserved, it might be considered that about an inch of the jaw had been destroyed.

Being obliged to accompany the Russian army, which retired from France, Mr. de R—, cured of his wounds, but not of his fracture, did not return here until 1815; and it was not until 1818 that he obtained permission to come to Paris, to seek a remedy for his infirmity. He was at this time in the following state:

Of the two fragments of the jaw, the posterior, constituted of what remained of the condyloid apophysis, or rising branch, of this bone, and by the most posterior part of the alveolar margin, had the form of an elongated and flattened cone: it had effected a slight rotatory motion from without inwardly, so that its superior margin, instead of being turned towards the superior molares, was directed towards the tongue, whilst it was altogether separated far from the anterior fragment. It supported the dens sapientiæ, the crown of which, from the oblique position of the bone, lay in a slanting direction, from within outwardly. In front of this tooth, a sharp point, below which nothing could be felt, indicated that a portion of the alveolar margin, nearly an inch in length, had remained continuous with the rest of this fragment, but that all that portion of the bone which had existed below this margin, between it and the angle of the jaw, had been destroyed.

The anterior fragment, constituted of the rest of the jaw, had undergone such a displacement, that its extremity, corresponding with the fracture, was carried outwardly, and below the point of the preceding fragment. On carrying the finger from before backwards along the base of the jaw, we arrived at the cicatrix over this bone, at the right side of the face; the projection formed by the end of the anterior fragment; and, beyond this projection, the vacancy resulting from the change of situation and loss of substance sustained by the posterior fragment, were very easily perceptible. The loss of substance of the bone, judging from the situation of the remaining teeth, must have been about an inch in extent, and it affected that part of the base of the jaw which comprised the alveolæ of the two first large molares. However, the parts had so approached each other, that, on examining the state of them in the interior of the mouth, the vacancy left by the loss of two large teeth and of their alveolæ could hardly be recognized; the second small molaris of the anterior fragment being almost in contact with the dens sapientiæ of the posterior fragment, the right half of the dental arcade only appearing shorter than the left. There resulted from this such a want of the natural relation between the margins of the superior and inferior jaws, that the left lateral incisor of the lower struck against the right middle incisor of the upper jaw. But when, on seizing the low jaw by the chin and drawing it into its natural direction, the right side of it became elongated, an interval of nearly an inch was produced between the dens sapientiæ and the next tooth on this side.

The consequences of this state of the parts were, that the production of articulated sounds was very difficult, and the mastication of solid aliments impossible; besides these, when the chin was not supported by a stiff cravat, it fell towards the right side, distorting the face, and being attended with a constant flow of saliva from the mouth, which was half open.

Mr. de R—, first consulted Prof. Percy, who formed the intention of cutting off the ends of the fragments of bone, and placing them in contact, with the expectation that they would then become consolidated; and, either with the object of becoming better assured of the state of the parts, or from the hope of being able to manipulate with more facility the posterior fragment, which the elevator muscles maintained immovable and pressed against the superior dental arcade, he was induced to have the last large molaris of the upper jaw drawn. As soon as this was done, the posterior fragment, having no longer any thing which sustained it, and giving way to the efforts of the muscles just named, rose more and more upwards, turning on its condyle, until the tooth which it contained was lodged in the space left by the removal of that in the upper jaw. The point of its angle now projected in the cheek at about the height of the superior dental arcade, and seemed to be immovable in this position. The interval which separated it from the anterior fragment appeared to be considerably increased from above downwards.

This occurrence aggravated the incommodious condition of the patient; and the parts were in this state when M. Percy advised him to consult M. Dupuytren.

Several surgeons had told the patient that serious hemorrhage would be the consequence of the operation above designated, and that no advantage could be derived from it, because it would be impossible to place, and to maintain, the two fragments in contact. The last opinion kept M. Dupuytren in suspense for some time; but the entreaties of the patient, supported by the advice of M. Percy, at length removed his irresolution.

The excision of the ends of the fragments, was the chief remedial measure, and this appeared to him to present no difficulty; but this was not the case with regard to the preservation of contact between the portions; and he also considered, that the portion he might remove would add to this difficulty. Being convinced, by many instances, that it was sufficient, in analogous cases, to remove the end of one of the fragments only to obtain the consolidation of fractures, he stopped at the idea of removing by excision the end of the posterior fragment, and only rasping that of the anterior portion. But, as above mentioned, the loss of substance sustained by the posterior fragment affected the part of this fragment corresponding with the base of the jaw, and that it had preserved a portion of the alveolar margin, whilst, on the contrary, that of the anterior fragment affected the alveolar margin, the part corresponding with the base of the jaw being preserved: whence it resulted, that, even though each of the two fragments were brought into its natural site, they could not be placed in immediate contact, since there would be between the points by which they terminated, the natural interval which separates the base of the jaw from the alveolar margin.

This consideration did not lead M. Dupuytren to resign his purpose, because he has seen, after several instances of amputation of the middle part of the jaw, an osseous production corresponding in all respects with the natural parts, extend from one branch of the jaw to the other, and replace so exactly the portion of bone removed by amputation, that the patients, when viewed externally, did not appear to have been deprived of the natural prominence of the chin.

The greatest difficulty which presented itself, was the preservation of the parts in such a situation, that no deformity might remain after the consolidation became effected. Before proceeding to the operation, M. Dupuytren wished to try the means he could employ for the fulfilment of this indication: to this effect, he used some mechanical apparatus, which was found to be ineffectual; though this advantage was derived from the trial, that the posterior fragment was rendered more easily movable than it had been previously. The failure of these measures rendered the case more and more embarrassing; when M. Lemaire, a surgeon-dentist, proposed a method which was both simple and appeared likely to be effectual. It consisted, 1, in replacing the superior molaris which had been drawn by one of ivory, which might oppose the ascension of the posterior fragment; 2, in maintaining the two fragments in a proper position by means of platina wire, attached, on the one part, to the teeth implanted in the fragments near the fracture, and, on the other, to the opposite teeth in the upper jaw. A previous trial of these measures having been made, M. Dupuytren, well persuaded that there was nothing dangerous in the operation, and that, if it failed, it would not render the state of the patient more disagreeable, proceeded to practise it in the following manner, on the 14th July, 1818.

The patient was seated in a chair. The operator grasped the thick part of the right cheek between the thumb and fore-finger of his right hand, whilst, with a bistoury held in the left, he divided the parts from without inwardly and perpendicularly to the base of the jaw, at the distance of about a quarter of an inch behind the summit of the point formed by the posterior fragment: the cutting edge of the instrument having been carried to the bone, and the soft parts enveloping the latter, internally as well as externally, having been divided by a circular incision, he substituted for the bistoury a very narrow saw, with which he effected the excision of a portion of the end of the posterior fragment, of a triangular shape, the point of which, obtuse and cicatrized, adhered to the soft parts of the cheek, and of which the base corresponding to the section which had been made, as well as the two sides which were obtuse and cicatrized like the point, was of about three inches in length.* This portion was extracted by the interior of the mouth.

M. Dupuytren then removed the soft parts from the end of the anterior fragment, by means of an instrument similar to the cutters used by wood-engravers, all but the internal part of the

* This is a verbal translation of the original; but there is, it would appear, some error in the account: that the operator removed a triangular portion of bone, each of the sides of which was three inches in length, does not seem probable. The description altogether is not very lucid or satisfactory; we shall therefore give the original, in this instance. "Le malade, étant placé sur une chaise, l'operateur saisit, entre le pouce appuyé sur la peau et l'indicateur de la main droite porté dans l'interieur de la bouche, l'epaisseur de la joue droite, tandis qu'avec un bistouri tenu de la main gauche, il traversa les parties de dehors en dedans et perpendiculairement à la base de la machoire, à peu près à trois lignes en arrière du sommet de la pointe formée par le fragment posterieur; le tranchant de l'instrument ayant été abaissé jusqu'à l'os, et les chairs qui recouvraient ce dernier, tant en dedans qu'en dehors, ayant été divisées circulairement, il substitua au bistouri une scie à manche et à lame très-étroite, avec laquelle il opéra la résection d'une portion osseuse, triangulaire, dont la point mousse, cicatrisée, adhérait aux parties molles de la joue et dont la base correspondant à la section qu'on varait faire, avait, ainsi que ses deux bords qui était emoussés et cicatrisés comme la pointe, environ trois pouces de longeur."—Edit.

gum, which formed a sort of ridge extending from the second molaris to the dens sapientiæ, and which was preserved as a barrier to prevent the saliva penetrating between the two fragments, and continually bathing their extremities.

The parts being thus prepared, and the anterior fragment reduced to its proper situation, M. Lemaire began by putting in the place of the last upper molaris a strong piece of the tooth of the hippopotamus, the upper surface of which was moulded to the gum, whilst its inferior surface presented a cavity to receive the dens sapientiæ of the lower jaw. This substance was fixed to the adjacent molaris by platina wire: the object of it was to replace the tooth which had been extracted, and to keep down the posterior fragment. A fold of the same wire was then twisted round the crown of the dens sapientiæ in the posterior fragment; and the two ends of it were carried across the mouth, above the tongue, to the crown of the small molaris of the left side, to which they were firmly attached. This wire was intended to draw the posterior fragment inwardly, and to maintain it thus in the same line as the anterior fragment.

Lastly, it was necessary to fix, for greater security, the two fragments, thus united, to the upper jaw. For this purpose, another piece of wire was passed from the first small molaris of the upper jaw to that of the lower, which were firmly attached together; but this was not effected without much difficulty: the muscles acted with such violence as to break the wire repeatedly, and two hours elapsed in the attempt. Another loop of wire, passed between the right lateral incisor and the canine teeth of the lower jaw, was twisted round the left superior canine tooth: the two jaws were brought into contact in such a way, that the left middle incisor of the lower jaw, instead of corresponding with that of the upper, answered to the right middle incisor of this jaw. This deviation from the natural position was made, from the fear that, with the great loss of substance, the placing the parts in their proper site would leave an interval between the fragments that no production would be able to fill up.

The ordinary chin-bandage was applied; the patient was placed in bed, and ordered to preserve absolute silence. He wrote what he wished to communicate. The constant use of pediluvia and glysters was directed; and, although at length he was advised to take a little nutriment, he never would, during the whole time of the cure, take any thing more than a cup of tea with milk in the morning, and a bason of broth in the course of the day.

The first night was passed very quietly; but some unpleasant things soon occurred. The pressure of the artificial tooth on the gum sustaining it, and that of the first wire on the tongue, produced extremely acute pain in these parts. The patient several times expressed his fear that his tongue was quite cut through. Besides this, the wires fixed to the upper teeth transmitted to these the efforts made by the fragments of bone to displace themselves; the consequence of which was, severe pains in the upper jaw, which recurred in paroxysms, sufficiently frequent to deprive the patient of sleep, and sufficiently severe to produce tears. He was also incommoded by a fatiguing cough, probably arising from the difficulty he had in removing the mucous fluids which collected about the fauces. There was, however, no fever, nor any pain in the part engaged in the operation.

On the eighth day, some intervals of ease began to become apparent. Some pus was mingled with the mucus which escaped from the mouth. An infusion of marsh-mallow roots was then injected into the mouth several times a-day; and this practice, being very agreeable to the patient, was continued to the end of the treatment.

The bandage was not renewed until the twenty-eighth day. The little exterior wound was then almost cicatrized. The flow of pus from the mouth, which had never been abundant, diminished. The pain in the tongue was less violent: that of the upper teeth returned only after long intervals, as, for instance, every other day. The pain occasioned by the pressure of the artificial tooth on the gum was continuous, and always very severe.

On the thirtieth day, although the wires were sufficiently tight to prevent any separation of the two jaws from each other, it was nevertheless observed that the chin had retreated directly backwards, so that the lower incisors, instead of being in contact with the posterior surface of the upper ones, were distant from a line and a half or two lines. This circumstance, which was not again observable on the thirtieth day, manifested itself several times since; and it was in this position that the cure was effected.

The bandage was renewed on the thirty-second day, in consequence of its having become a little too lax; and the patient had suffered very severe pain in the preceding night. The wound in the cheek was found entirely cicatrized. The pains ceased. That of the tongue disappeared on the fortieth day, as well as that affecting the upper teeth. The flow of pus from the mouth was stopped. The absence of pain seemed to show that the fragments had no longer any disposition to become displaced, and the absence of pus, that the interior wound had healed. But at this time acute pain was experienced in both ears, especially in the right. This pain, after having once disappeared from the forty-eighth to the fiftieth day, did not totally cease until the fifty-second day.

On the sixty-first day the bandage was removed, in order that the state of the parts might be better examined. Twenty days had now elapsed, during which the patient had suffered no other pain than that from the pressure of the artificial tooth on the gum supporting it. For the same time the lower incisors had remained completely hidden behind the upper ones, and were situate a little more backwardly than in the natural state, so that the lower jaw altogether seemed to have effected a slight movement backwards.

The finger, carried along the base of the jaw, encountered, under the skin, the point of the anterior fragment; but, at the same time, it detected a new-formed production, which was firm, resistent, and which manifestly extended from below upwards, and from before backwards, from the anterior to the posterior fragment. This state of things being as satisfactory as possible, it was determined that two days hence some portion of the apparatus should be removed. On the sixty-third, the two pieces of wire which fixed one jaw to the other were taken away. Some slight movements of elevation and depression of the jaw were then produced; and, though it caused some regret to find that it inclined a little to the right side when depressed, it was observed, with not less astonishment, that the patient had recovered the power of bringing it to its proper position, on closing it; so that the movements it performed exactly resembled those of mastification of an herbivorous animal. The removal of the rest of the apparatus was deferred to another period.

On the sixty-eighth day it was determined that the wire which traversed the mouth should be removed, from the consideration that the time necessary for the re-union of the fracture being expired, no hope of its ever being effected could be entertained, if it had not taken place at this period; and that the inclination of the jaw towards the right side when depressed was no proof of the want of consolidation of the fracture, since some shortening of this side of the jaw might be expected to exist; and it might be manifested only when the jaw was depressed, because the condyle was drawn a little from its proper place by the apparatus, whilst it returned to it when this was removed and the jaw a little depressed. The fear expressed by the patient that his tongue was cut through, was not without foundation; and the circumstance was explicable by this removal and depression of the position of the condyle. It was now discovered that the ends of the loop of wire which crossed the mouth had cut through half the thickness of the tongue; but, as the surface first divided united as the wires penetrated deeper, these were found situate in the middle of the tongue, traversing it through the middle from one side to the other. They were cut through on each side near to the teeth, and the portions in the tongue then withdrawn.

The patient was now permitted to speak, and he was directed a more nutritious diet; but the artificial tooth was not removed until fifteen days afterwards. Six days after this, when he came to visit M. Dupuytren, it was already observable that the jaw turned less to the right side when depressed than it had done a short time previously.

Three months after the operation, when Mr. de R— left Paris, his face had recovered its symmetry; the chin occupied its proper position; the teeth of the lower jaw, placed behind those of the upper, corresponded with them within about the thickness of an incisor tooth; the lower teeth could also be applied exactly against the upper. In the movement of depression, the jaw still inclined a little to the right, but, on being elevated, it recovered its ordinary position: the latter movement, marked, when the patient wished it, by a clashing of the teeth of this jaw against those of the upper, announced at the same time their direct encounter, and the strength of the elevator muscles. The articulation of sounds was clear and distinct; and the patient already made use of firm and solid aliments. A most careful examination left no doubt respecting the reality of the consolidation, which the circumstances above mentioned indicated in a positive manner; the posterior fragment, however, was still elevated a little above the anterior. Several letters have since been received from Mr. de R—, as well as from Count Woronzoff, by M. Dupuytren, which confirm the solidity of the cure.—*Journal Universel des Sciences Medicales*, Juillet 1820.

